# Accuracy of Patient Setup Using Surface Guided Radiotherapy (SGRT) for Abdominal Malignancies

**DOI:** 10.3390/mps8050119

**Published:** 2025-10-03

**Authors:** Varvara Sotiropoulou, Stefanos Kachris, Michalis Mazonakis

**Affiliations:** 1 Department of Medical Physics, Faculty of Medicine, University of Crete, 71003 Heraklion, Greece; 2 Department of Radiotherapy, University Hospital of Heraklion, 71110 Heraklion, Greece

**Keywords:** Surface Guided Radiation Therapy (SGRT), abdominal radiotherapy, patient setup accuracy, tattoo/laser positioning, Cone beam CT (CBCT) verification, axis-specific analysis, image-guided radiotherapy (IGRT)

## Abstract

This study aimed to evaluate the placement accuracy and reproducibility of Surface Guided Radiotherapy (SGRT) compared with the conventional tattoo/laser method in patients undergoing radiotherapy for abdominal malignancies. A retrospective analysis was conducted on 43 patients treated with either SGRT (Group A) or the tattoo/laser technique (Group B). Patients in both groups underwent CBCT to quantify the positioning shifts in the vertical (*Svrt*), lateral (*Slat*) and longitudinal (*Slng*) axes, as well as the total shift. Statistical indicators including median, interquartile range (IQR), and range were calculated, and Mann–Whitney U tests were performed due to non-normal data distribution. Median values in all axes were same between groups: *Svrt* = 0.4 cm, *Slat* = 0.2 cm, *Slng* = 0.4 cm. Group A showed a higher total median shift equal to 0.8 cm versus 0.7 cm of Group B. However, IQRs were smaller in the Group B for all directions and total shift, indicating greater method consistency. Statistically significant differences (*p* < 0.05) were observed in all axes, except the vertical. These findings suggest that, while SGRT achieves comparable median alignment, its use in a highly variable anatomical region such as the abdomen may be associated with greater setup variability.

## 1. Introduction

Radiotherapy (RT) is one of the main treatment techniques for cancer, offering highly effective local control for a wide range of malignancies. The pillar of radiotherapy is directly linked to the ability to deliver high doses to the target volume while simultaneously minimizing the dose to surrounding healthy tissues and organs at risk (OARs). Inaccuracies in patient positioning may cause adverse effects, such as suboptimal dose delivery to the tumor and unnecessary radiation exposure to healthy structures, potentially increasing treatment-related toxicity.

The abdomen is particularly prone to setup variability, as it is affected by factors such as breathing, bowel or gastric volume, and overall patient relaxation. This anatomical variability emphasizes the need for highly accurate and reproducible patient positioning to ensure that the prescribed dose is accurately delivered to the tumor while minimizing the dose to surrounding healthy tissues. Abdominal tumors are surrounded by critical radiosensitive organs including kidneys, liver, bowel, spinal cord and stomach. Possible overexposure of these healthy structures and exceedance of the dose tolerances of the above normal tissues may lead to serious complications such as renal dysfunction, hepatic failure, bowel obstruction, ulceration and/or myelitis/necrosis [1,2].

The conventional method for patient positioning relies on external skin tattoos and laser-based alignment. While this method is widely adopted, it is subject to inherent limitations, including skin deformation, anatomical changes, and patient discomfort related to the use of permanent tattoos [3,4,5,6]. In recent years, SGRT has emerged as a modern technique for patient setup prior to therapeutic irradiation. It utilizes multiple stereoscopic camera units combined with structured visible light to capture the patient’s surface in real time [7].

Several studies have evaluated SGRT across various anatomical sites and found that it significantly improves setup accuracy compared to conventional tattoo/laser alignment [3,4,5,6,7,8,9,10,11,12,13,14]. For instance, Rudat et al. [14] analyzed 2303 fractions across 183 patients (including abdominal cases) and reported a mean 3D setup deviation of 3.6 mm (95% confidence interval (CI) 3.3 mm to 3.9 mm) and 4.5 mm using laser alignment with skin marks (95% CI 3.9 mm to 5.2 mm; *p* = 0.001). However, there is much consideration regarding the application of SGRT in patients suffering from abdominal malignancies, as surface deformities affect the reliability of SGRT in this region [15]. While studies in thoracic and pelvic regions report consistent SGRT accuracy, the abdomen remains under-investigated, and some findings even highlight larger residual errors in abdominal SGRT setups [15].

The purpose of this study was to evaluate the accuracy of SGRT-based patient setup compared to the conventional method specifically in the abdominal region, using Cone beam Computed Tomography (CBCT)-based positioning corrections as a reference. By analyzing the 3D shifts across the different anatomical axes (*Svrt*, *Slat*, *Slng*), this study aims to provide further insight into the clinical performance of SGRT in a challenging anatomical site where data remain limited.

## 2. Materials and Methods

### 2.1. Patients’ Group

#### 2.1.1. Patient Cohort and Clinical Characteristics

The current study took place in the Radiotherapy Department of the University General Hospital of Heraklion. A total of 43 patients who underwent RT for several abdominal malignant diseases and received varying tumor dose levels were retrospectively included in this study. The cohort was divided into two groups based on the method of positioning: SGRT (Group A) and conventional tattoo-based setup (Group B). For both groups, the number of patients per malignant disease is shown in Table 1. The conventional technique of three-dimensional conformal radiotherapy (3D-CRT) or the modern techniques of intensity modulated radiotherapy (IMRT) and volumetric modulated arc therapy (VMAT) were applied for treating the abdominal tumors of the study participants. The prescribed tumor dose in these patients varied from 5 Gy to 55 Gy depending upon the conditions of the malignant disease under treatment.

Contouring of both target volumes and organs at risk (OARs) followed standardized ESTRO (European Society for Radiotherapy and Oncology) guidelines to ensure consistency, accuracy, and reproducibility in delineation across all cases [16,17,18]. Organs at risk included the kidneys, liver, spinal cord, bowel, stomach and spleen (in cases where it was not overlapping with or included in the target volume).

The patient’s allocation was determined by treatment period: Group A consisted of all patients who underwent abdominal radiotherapy in our department between July 2023—when SGRT was implemented—and January 2025. Group B included patients treated for abdominal tumors in the two years immediately preceding SGRT availability. For purposes of comparability, the patients belonging to Group B patients were selected to have similar malignant diseases—upper gastrointestinal tumors, lymphomas, and sarcomas—with those of patients in Group A.

##### Group A

Nineteen patients (11 males and 8 females), aged between 39 and 88 years (median age: 72 years, mean age: 69.2 years), were positioned on the linear accelerator couch using the SGRT approach. Eight patients in this group had hematologic malignancies. Six were diagnosed with non-Hodgkin lymphoma (NHL): four received RT for residual disease evident on PET-CT following chemotherapy, where local control was considered critical to prevent further systemic progression, while two received consolidation RT after achieving partial remission, consistent with current practice guidelines for high-risk NHL. One pa-tient with bulky Hodgkin disease underwent RT as part of combined-modality therapy, where localized irradiation remains an essential component to reduce relapse risk in large mediastinal or abdominal masses. Another patient, previously treated for Hodgkin lym-phoma, received palliative splenic irradiation for symptomatic splenomegaly, with the aim of alleviating pain, cytopenias, and pressure symptoms as shown in Table 1.

Five patients were treated for upper gastrointestinal (UGI) cancers. Two patients re-ceived adjuvant RT following suboptimal (less than D_2_) gastrectomy, where locoregional recurrence risk is high and RT can improve disease control. One patient with advanced gastric cancer underwent palliative RT to reduce bleeding and alleviate tumor-related ob-struction. Another patient with an esophagogastric junction tumor received definitive RT, as surgical resection was not feasible due to comorbidities and disease extent. One patient with pancreatic adenocarcinoma was treated with adjuvant RT after resection, where the intent was to enhance local control given the high rate of locoregional recurrence.

Additionally, two patients underwent preoperative RT for intra-abdominal leiomyo-sarcomas. The rationale was to improve surgical resectability and achieve better local control, as sarcomas often present with large, marginally resectable tumors. Four additional patients received RT for metastatic para-aortic lymph node involvement: three originating from uterine carcinomas, where nodal irradiation is recommended to improve disease control and symptom palliation, and one from prostate cancer, where RT was ap-plied with palliative intent to control nodal progression and related symptoms.

##### Group B

Twenty-four patients were treated with conventional tattoo-based setup. The median age was 59 years (range: 16–84 years, mean age: 59.1 years), with a predominance of male patients (19 males and 5 females).

Ten patients were diagnosed with hematologic malignancies. Nine had NHL: five received consolidation RT to sites of bulky or initially involved disease, in accordance with the role of RT in consolidating systemic treatment responses, while four were treated for residual disease after chemotherapy, where RT offers durable local control. One patient with bulky Hodgkin disease received RT as part of combined-modality therapy, where ir-radiation of residual mass after chemotherapy remains standard in selected high-risk cases.

Twelve patients presented with UGI malignancies. Nine were irradiated for gastric cancer following suboptimal surgical resection, as locoregional recurrence is common when lymphadenectomy is inadequate. Among these, one received palliative RT to ad-dress tumor-related bleeding and pain. Two patients with pancreatic adenocarcinoma underwent chemoradiotherapy following induction chemotherapy, reflecting the strategy of integrating RT for patients with locally advanced disease not amenable to surgery but without distant progression.

Lastly, two patients underwent neoadjuvant RT for retroperitoneal sarcomas, a set-ting in which RT is frequently recommended to improve the probability of achieving neg-ative surgical margins and to enhance long-term local control.

### 2.2. Planning CT Scanning and Radiation Therapy

A planning computed tomography (CT) scan was performed using a 64-slice GE Revolution GSI scanner (GE Healthcare, Chicago, IL, USA), covering the entire abdominal region. The participants were scanned in the treatment position. All patients were supine during scanning with both arms positioned alongside the head, with the knees slightly flexed. Immobilization devices were used to stabilize the legs and the arms and minimize pelvic or abdominal rotation. The CT images were transferred to the Monaco treatment planning system (Elekta AB, Stockholm, Sweden). A senior radiation oncologist manually delineated the target volume, organs-at-risk and external body contour on an image-by-image basis was defined with the appropriate software tools. Regarding the external contour, the region of interest (ROI) was carefully selected to include stable external surfaces, particularly the anterior abdominal wall, and exclude variable regions such as the upper abdomen and the legs, following ESTRO-ACROP guidelines [7].

Therapeutic irradiations were delivered using the following medical linear accelerators installed in our department:

- Linac 1 Infinity (Elekta, SW): 6 MV single photon energy and a CBCT system XVI R5.0.4 (Elekta, SW). All patients of Group B were treated on this machine.
- Linac 2 Infinity (Elekta, SW): 6, 10, 15 MV photon energies and a CBCT system XVI R5.0.4 (Elekta, SW). All patients of Group A were treated on this machine.

### 2.3. Patient Setup Method

For the Group B, three permanent skin marks were applied on the patient’s body during the planning CT scan to define a transverse reference plane, with two marks on the lateral sides and one on the anterior surface. These marks were aligned with the room lasers, which indicate the system’s isocenter, to guide daily patient setup. For all patients of Group B, the setup was performed manually using the permanent tattoos aligned with room lasers. The daily quality control of the therapy machine always included the check of the alignment of the in-room lasers [19].

The setup of all patients including in Group A was performed using the AlignRT surface guidance system (Vision RT, London, UK). The external contour of the patient’s body, as defined in the Monaco system, was imported into the software version 6.3 of AlignRT system. This contour was taken as the reference surface for optical tracking. The AlignRT system uses three ceiling-mounted pods, each equipped with stereoscopic cameras and a structured light projector, to capture the patient’s real-time surface and compare it to the reference surface derived from the planning CT. The software calculates six degrees of freedom (DOF) real-time deltas (RTDs), reflecting translational (*vrt*, *lat*, *lng*) and rotational (pitch, roll, yaw) deviations. Radiation therapists manually adjusted the couch position in all six DOFs to ensure that the RTDs were within predefined clinical tolerances before proceeding to treatment [7,13].

It should be mentioned that the performance of the SGRT system installed in our department was periodically checked. A specially designed calibration plate consisting of an array of high-contrast circular black dots on a white flat surface was employed for the system calibration [20]. The above plate was placed on the couch of the linear accelerator and its center was aligned with the isocenter of the therapy machine during this procedure [21]. The isocenter of the imaging system was determined on the basis of this calibration procedure. The above calibration of the system was carried out by the medical physicists of the department every month. Furthermore, daily checks were performed before the clinical use of the SGRT system to ensure the integrity of each camera pod placed on the ceiling of the treatment room.

### 2.4. CBCT Verification and Position Correction

After the initial setup, patients in both groups underwent CBCT imaging using a scanning system (Elekta XVI R5.0.4) integrated to the aforementioned Linacs 1 and 2. CBCT imaging was used for position verification and correction. The rotation axis of the XVI was coincident with the axis of the source emitting the therapeutic photon beams. The CBCT system utilizes an amorphous silicon (*a*Si)/cesium iodine (CsI) flat-panel detector. The system also has an X-ray source which operates at 80 to 140 kV. The size of the radiation field emitted by the source of the XVI system may be chosen with the aid of cassettes having standard openings. Images can be obtained with three different field-of-views (FOV) denoted as small, medium and large. The medium FOV was selected for the acquisition of all abdominal images. A radial field of view of 41.0 cm at the plane of the detector with a length of 27.7 cm was applied. The other acquisition parameters for the abdominopelvic protocol implemented for adult patients were the following: 120 kVp, 1.6 mAs per frame, 660 frames, 360° angular span, −180° start angle and rotation in clockwise direction. All CBCT scans were acquired using a bow-tie filter. The above scanning parameters have been suggested by the vendor of the linear accelerators of our department for male and female patients treated for abdominoplevic malignancies.

For VMAT treatments, CBCT was performed daily, whereas for 3D-CRT treatments, it was performed during the first three sessions and subsequently every five sessions. The CBCT image was displayed on the system’s console and overlaid with the pre-treatment planning CT image to identify any positional discrepancies. The goal of this image matching (registration) process was to achieve full alignment between the patient’s current anatomy (CBCT) and the reference planning anatomy (CT). Registration was performed using visible anatomical landmarks, including soft tissue structures or bony anatomy. Once registration was complete, the system automatically calculated and applied the necessary couch shifts to correct the patient’s position in the vertical (*Svrt*), lateral (*Slat*), and longitudinal (*Slng*) directions. Although the AlignRT system provided information on both translational and rotational deviations in six degrees of freedom, the couch corrections applied after CBCT verification were limited to translational adjustments only. These corrections, measured in centimeters, were recorded and stored by the system for each treatment session.

The total displacement (*Stot*) for each fraction was calculated based on the shift values *Svrt*, *Slat*, and *Slng* recorded for that specific fraction, using the following Formula:

$$Stot=\;\sqrt{{Svrt}^{2}+{Slat}^{2}+S{lng}^{2}}$$

### 2.5. Statistical Analysis

Microsoft Excel was used to record and classify the data. Data processing and all statistical analyses were performed using Python (version 3.11.9). For each axis (*Svrt*, *Slat*, *Slng*) and the total displacement, median values and IQRs were calculated, while visualizations (strip plots and Box and Whisker plots) were generated using seaborn and matplotlib libraries. Outliers were defined according to the conventional 1.5 × IQR criterion and are displayed as individual points in the box-and-whisker plots. No removal or transformation of outlier values was performed, as these measurements were considered potentially relevant to real-world positioning variability.

Normality tests were conducted using the Shapiro–Wilk test. As all datasets were not normally distributed (*p* < 0.05), comparisons between the groups A and B for each pair of displacements were performed using the non-parametric Mann–Whitney U test. A 95% confidence level was applied, and *p*-values below 0.05 were considered statistically significant.

## 3. Results

### 3.1. Patient Positioning Corrections

Patient positioning corrections were analyzed and compared between groups A and B. Group A consisted of a total of 164 treatment fractions, resulting in 164 recorded shifts in the vertical, lateral, and longitudinal directions, as well as 164 corresponding total shifts. Group B included 198 treatment fractions, producing 198 shifts per axis and 198 total shifts. Table 2 summarizes, for each axis and method, the median shift and the range of the measured shifts (minimum–maximum values).

**Figure 1:**
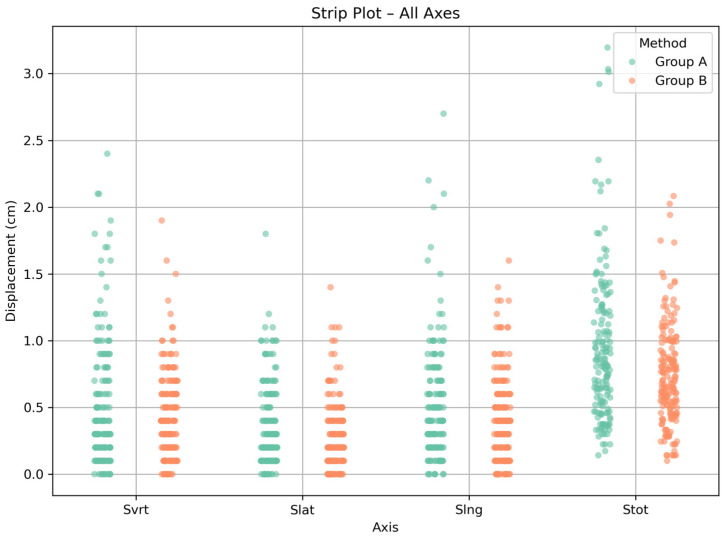
Strip plot showing the distribution of setup shifts per axis (*Svrt*, *Slat*, *Slng*, *Stot*) for both Group A and Group B methods across all treatment fractions. Each point represents the displacement value for an individual session.

The median shift values were 0.4 cm in the vertical direction, 0.2 cm in the lateral direction, and 0.4 cm in the longitudinal direction for both groups. The total 3D shift had a median value of 0.8 cm for Group A and 0.7 cm for Group B. Regarding the range of values, Group A showed displacements from 0.0 to 2.4 cm (*Svrt*), 0.0 to 1.8 cm (*Slat*), 0.0 to 2.7 cm (*Slng*), and 0.1 to 3.2 cm (*Stot*). For Group B, the corresponding ranges were 0.0 to 1.9 cm (*Svrt*), 0.0 to 1.4 cm (*Slat*), 0.0 to 1.6 cm (*Slng*), and 0.1 to 2.1 cm (*Stot*). Figure 1 displays a strip plot showing the distribution of individual shift values per axis for all treatment fractions in both groups.

### 3.2. Statistical Comparison

The Shapiro–Wilk test was applied to assess the normality of each dataset. All *p*-values were <0.001, indicating that the distributions were not normal and non-parametric testing was required. Mann–Whitney U tests were conducted to compare the distributions between groups. Statistically significant differences (*p* < 0.05) were found for the lateral, longitudinal, and total shift values, while no significant difference was observed in the vertical axis (*p* = 0.31). Table 3 summarizes, for each axis and method, the interquartile range (IQR) and the first (Q1) and third (Q3) quartiles. Q1 and Q3 represent the 25th and 75th percentiles of the shift values, respectively.

**Figure 2:**
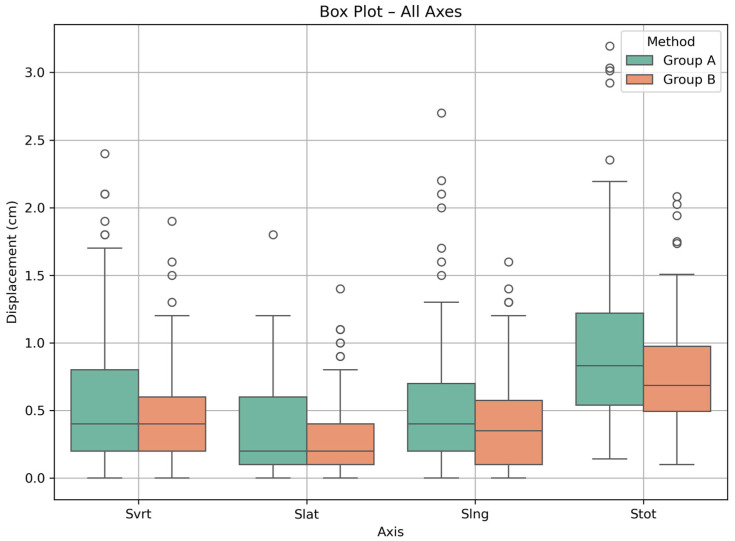
Box plot illustrating the distribution of setup shifts per axis (*Svrt*, *Slat*, *Slng*, *Stot*) for Group A and Group B.

Interquartile ranges (IQRs) for Group A were 0.2–0.8 cm (*Svrt*), 0.1–0.6 cm (*Slat*), 0.2–0.7 cm (*Slng*), and 0.5–1.2 cm (*Stot*). For Group B, the IQRs were 0.2–0.6 cm (*Svrt*), 0.1–0.4 cm (*Slat*), 0.1–0.6 cm (*Slng*), and 0.5–1.0 cm (*Stot*). Figure 2 presents box plots visualizing the spread and central tendency of displacements per axis for both groups.

## 4. Discussion

Cone-beam computed tomography is widely used in several clinical fields such as dentistry, orthodontics, otolaryngology and maxillofacial surgery [22,23,24,25,26]. Moreover, CBCT is currently considered as a valuable tool for image guidance of patients prior to and during radiation therapy [27]. Modern linear accelerators generating therapeutic megavoltage beams are equipped with CBCT systems enabling kV imaging. The aim of this study was to evaluate the accuracy of SGRT technique compared to the conventional tattoo/laser method in abdominal radiotherapy. The comparison of the above methods was based on the results obtained by CBCT examinations of the study participants subjected to therapeutic irradiation for tumors in the abdomen. The abdominal region presents high variability between patient radiotherapy sessions due to factors such as respiratory movement, bowel filling, etc., making accurate and reproducible patient positioning a critical requirement for optimal dose delivery to the target tumor, but also for the protection of OARs.

The results of this analysis showed that the median shift values between the two groups were identical across all three anatomical axes, measured at 0.4 cm, 0.2 cm, and 0.4 cm for the *vrt*, *lat* and *lng* axes, respectively. For patients of Group A subjected to SGRT, the median value of the total shift was found to be 0.8 cm. This value was slightly higher than that calculated for patients of Group B whose setup was based on the conventional approach of laser and tattoos. At first glance, these findings may suggest comparable positioning accuracy between the two methods when only the central tedency of the shifts for each axis are considered.

However, in a more extensive analysis of the results and specifically by observing the IQRs, differences were revealed in the spread of the data. For all axes as well as for the total shift the IQRs were smaller in the Group B, indicating less variability among patients. Specifically, in the vertical axis, Group A had an IQR equal to 0.6 cm (Q1 = 0.2, Q3 = 0.8), compared to 0.4 cm (Q1 = 0.2, Q3 = 0.6) in the Group B. In the lateral direction, the IQRs for Group A were 0.5 cm (0.1–0.6) versus 0.3 cm (0.1–0.4) for Group B, and in the longitudinal direction, both methods had an IQR of 0.5 cm, although SGRT showed a wider range overall. For the total shift, SGRT had an IQR of 0.7 cm (0.5–1.2), compared to 0.5 cm (0.5–1.0) for the laser/tattoo group.

For all comparisons, non-parametric Mann–Whitney U test results were also extracted, showing significant differences (*p* < 0.05) on all axes except the vertical axis, where the *p*-value was greater than 0.05. This indicates that while the median values may appear similar between methods, the Tattoo/Laser technique yielded more consistent adjustments in most directions, potentially reducing the risk of large deviations in daily practice.

Although SGRT offers several advantages, such as being non-invasive, eliminating permanent skin marks, and enhancing patient comfort, its application in abdominal radiotherapy still remains under question. This is due to the deformable nature of the abdominal surface, as well as its susceptibility to breathing-related motion and internal organ shifts, which can impair the accuracy of surface registration. These challenges may partly explain the increased variability observed in SGRT positioning in this study.

Psarras et al. [15] reviewed the results of previous studies comparing the two techniques for the positioning of patients with abdominal tumors [28,29,30], noting that Carl et al. [29] and Walter et al. [28] assessed the feasibility of SGRT in the abdominal region through theoretical or planning-based simulations, without incorporating real-time clinical data or daily setup measurements. In contrast, the present study offers empirical results based on actual displacement data collected over multiple treatment fractions, providing a more realistic view of SGRT’s performance in clinical conditions. Nevertheless, both Carl et al. [29] and Walter et al. [28] observed that SGRT tends to result in increased displacement values and concluded that the tattoo/laser technique may offer more consistent positioning. This is in agreement with our findings, where SGRT showed greater variability, as reflected in the wider interquartile ranges across all directions.

Rudat et al. [14] reported lower mean 3D residual setup errors using SGRT compared to tattoo-based alignment in abdominal cases, suggesting improved accuracy. However, their analysis was limited to total shift deviations, without examining individual anatomical directions. Our findings complement this work by providing axis-specific comparisons and revealing that, despite similar median shifts between methods, the SGRT group exhibited greater variability across all directions. This suggests that in abdominal setups, SGRT may be more sensitive to internal motion and anatomical changes.

This study also has several limitations. First, the patient sample studied consisted of patients with various abdominal malignancies (e.g., hematological, upper gastrointestinal, and sarcomas), which may affect the reproducibility of the preparation due to differences in target location and internal anatomy through which the shifts arise. Second, the sample studied was small as it consisted of patients who underwent radiotherapy exclusively in our department at the University General Hospital of Heraklion. This may affect the statistical power of the study, and for more reliable results, a larger sample is needed. Another limitation of this study is the absence of recorded data on patient setup time, which could have provided additional insight into the practical efficiency of each method. While previous studies in other anatomical regions have reported mixed results, SGRT has been associated with reduced imaging or setup time in the head and neck area [31,32] and a 15% shorter setup time in thoracic radiotherapy [33]. In the abdominal region, however, evidence remains limited. A prior study conducted by our group [3] found that SGRT required longer setup times compared to tattoo/laser positioning. Future prospective studies should include timing metrics to better evaluate both the accuracy and workflow efficiency of SGRT in abdominal treatments.

## 5. Conclusions

In conclusion, while SGRT and tattoo-based setups achieved similar median shifts, the Tattoo/Laser method exhibited less variability, which may be advantageous in clinical settings where consistency and reproducibility are essential. These results highlight the importance of considering not only average accuracy but also intrafractional variability when evaluating setup methods. Further studies with larger sample sizes and advanced surface matching protocols are needed to improve SGRT reliability in this anatomically complex region.

## Figures and Tables

**Table 1:** Patient characteristics stratified by positioning method and disease type:.

Disease Category	Subtype	Group A (*n* = 19)	Group B (*n* = 24)
Hematologic Malignancies	Non-Hodgkin Lymphoma (NHL)	6	9
	Hodgkin Disease/Splenomegaly	2	1
	Para-aortic Metastases	4	0
Upper GI Cancers	Gastric Cancer	3	9
	Pancreatic Adenocarcinoma	1	2
	Esophagogastric Junction (EGJ)	1	0
Sarcomas	Intra-abdominal/Retroperitoneal	2	2

**Table 2:** Descriptive statistics (median and range) results for the shifts per axis and method.

Setup Technique	Displacement	Median (cm)	Range (cm)
Group A	*Svrt*	0.4	0.0–2.4
	*Slat*	0.2	0.0–1.8
	*Slng*	0.4	0.0–2.7
	*Stot*	0.8	0.1–3.2
Group B	*Svrt*	0.4	0.0–1.9
	*Slat*	0.2	0.0–1.4
	*Slng*	0.4	0.0–1.6
	*Stot*	0.7	0.1–2.1

**Table 3:** Descriptive statistics of setup shifts per axis and method, including the IQR and the first (Q1) and third (Q3) quartiles.

Setup Technique	Displacement	IQR (Q1, Q3) (cm)	Q1 (cm)	Q3 (cm)
Group A	*Svrt*	0.6	0.2	0.8
	*Slat*	0.5	0.1	0.6
	*Slng*	0.5	0.2	0.7
	*Stot*	0.7	0.5	1.2
Group B	*Svrt*	0.4	0.2	0.6
	*Slat*	0.3	0.1	0.4
	*Slng*	0.5	0.1	0.6
	*Stot*	0.5	0.5	1.0

## Data Availability

The data presented in this study are available on request from the corresponding author.
